# Activity Recognition and Semantic Description for Indoor Mobile Localization

**DOI:** 10.3390/s17030649

**Published:** 2017-03-21

**Authors:** Sheng Guo, Hanjiang Xiong, Xianwei Zheng, Yan Zhou

**Affiliations:** State Key Laboratory of Information Engineering in Surveying, Mapping and Remote Sensing (LIESMARS), Wuhan University, Wuhan 430072, China; guogis@126.com (S.G.); 2009302590044@whu.edu.cn (Y.Z.)

**Keywords:** activity recognition, indoor localization, semantics

## Abstract

As a result of the rapid development of smartphone-based indoor localization technology, location-based services in indoor spaces have become a topic of interest. However, to date, the rich data resulting from indoor localization and navigation applications have not been fully exploited, which is significant for trajectory correction and advanced indoor map information extraction. In this paper, an integrated location acquisition method utilizing activity recognition and semantic information extraction is proposed for indoor mobile localization. The location acquisition method combines pedestrian dead reckoning (PDR), human activity recognition (HAR) and landmarks to acquire accurate indoor localization information. Considering the problem of initial position determination, a hidden Markov model (HMM) is utilized to infer the user’s initial position. To provide an improved service for further applications, the landmarks are further assigned semantic descriptions by detecting the user’s activities. The experiments conducted in this study confirm that a high degree of accuracy for a user’s indoor location can be obtained. Furthermore, the semantic information of a user’s trajectories can be extracted, which is extremely useful for further research into indoor location applications.

## 1. Introduction

Location-based services (LBSs) have been popular for many years. Although global navigation satellite systems (GNSSs) can provide good localization services outdoors, there is still no dominant indoor positioning technique [1]. Therefore, an alternative technology is required that can provide accurate and robust indoor localization and tracking. Moreover, the spatial structures of indoor spaces are usually more complex than the outdoor environment, and thus, distinctive information is needed to better describe locations for the LBS-based applications.

With the wide availability of smartphones, a large amount of research has been conducted in recent years targeting indoor localization. Most of the existing indoor localization technologies require additional infrastructure, such as ultra-wideband [2], laser scanning systems (LSSs), radiofrequency identification (RFID) [3] and Wi-Fi access points [4]. However, these approaches often require extensive labor and time. To solve this problem, pedestrian dead reckoning (PDR) has recently been proposed as one of the most promising technologies for indoor localization [5]. Differing from the above approaches, PDR uses the built-in smartphone inertial sensors (accelerometer, gyroscope and magnetometer) to estimate the position. However, PDR suffers from error accumulation when the travel time is long. To achieve improved localization results, a number of studies have been conducted under particular circumstances, but the applicability and accuracy are still limited.

In addition to the direct application in indoor localization, the built-in smartphone sensors can also be used to understand the user’s movements [6], as well as to identify the indoor environment. Sensing the implied location information about the user moving in the corresponding environment provides a new opportunity for indoor mobile localization. To exploit this underlying information, some studies have been presented based on human activity recognition (HAR) [7,8,9], which uses these sensors to identify user activity and then infers information about the context of the user’s location. Therefore, it is worth exploring how to use this information to assist with indoor localization.

Recently, semantic information in the indoor environment has received increased attention. In many cases, semantic information is as valuable as the location. For example, from a human cognition perspective, in comparing the position coordinates, it is more valuable to know if a location is a room, a corridor or stairs [10]. Furthermore, it is also more convenient for a user to obtain semantic information (e.g., “turn left”, “turn right”, “go upstairs”, “go downstairs” and “go into a room”) than information about a route. However, the extraction and description of the necessary semantic information remains an open challenge.

In this paper, a method that combines PDR, HAR and landmarks is developed to accurately determine indoor localization. The proposed method requires no additional devices or expensive labor, and the user trajectory can be corrected and displayed. In addition, to solve the initial position determination problem, a hidden Markov model (HMM) that considers the characteristics of the indoor environment is used to match the continuous trajectory. Furthermore, to describe the user’s indoor activities and trajectories, an indoor semantic landmark model is also constructed by detecting the user’s activities.

**Figure 1:**
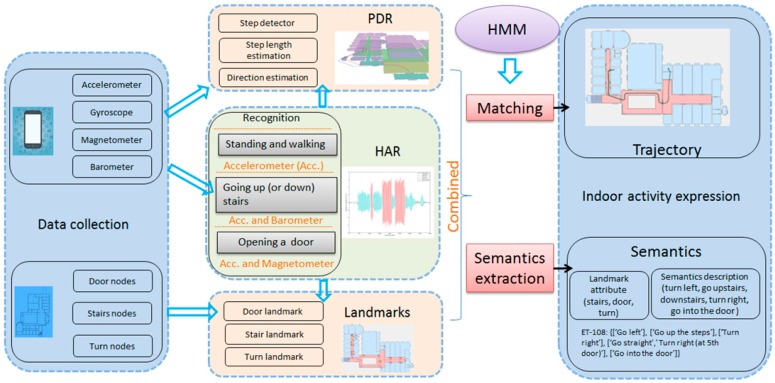
The overall architecture. HAR, human activity recognition.

Figure 1 shows an overview of the proposed approach.

The remainder of the paper is organized as follows. The related works are briefly reviewed in Section 2. The primary methods are then introduced in Section 3. Section 4 presents the experimental process, and Section 5 discusses and analyzes the experimental results. Finally, the conclusions and recommendations for future work are presented in Section 6.

## 2. Related Works

Most of the existing indoor localization technologies require additional infrastructure or expensive labor and time. How to achieve reliable and accurate localization in indoor environments at a low cost is still a challenging task [11]. Compared to other methods of indoor localization, using the built-in smartphone sensors provides a more convenient and less expensive indoor localization method, which has the advantage of providing continuous localization across the whole indoor space [12]. Smartphone-based pedestrian dead reckoning (SmartPDR) [13] has been the subject of increased attention, and it is now considered a promising technology for low-cost and continuous indoor navigation [1]. However, a number of parameters, such as step length and walking direction, can easily affect PDR’s localization accuracy [14]. Furthermore, PDR suffers from error accumulation over time because of the low-cost sensors, and thus, it is necessary to combine it with other methods, such as Wi-Fi, indoor map assistance or landmark matching.

With Wi-Fi routers widely deployed in most buildings [15], many studies have combined Wi-Fi and PDR to complement the other’s drawbacks. An early attempt can be found in [16], in which a PDR-based particle filter was used to smooth Wi-Fi-based positioning results, and a Kalman filter based on Wi-Fi was used to correct the PDR errors. Furthermore, a barometer was used to identify upstairs and downstairs. Another study [17] used a Bayes filter to combine PDR and Wi-Fi fingerprinting, and PDR was used to update the motion model. The Wi-Fi fingerprinting method was used to correct the model. In order to improve the efficiency of Wi-Fi-based indoor localization, some new techniques have been recently adopted, such as the Light-Fidelity-assisted approach [18] and received signal strength estimation based on support vector regression [19].

Aside from Wi-Fi, indoor maps can also be used to reduce the accumulated error of PDR. A number of studies have used map information for location correction. In [20], the user’s location, stride length and direction were used as the state values of the particle filter. To achieve localization with less computational resources, a conditional random field (CRF)-based method was proposed in [21]. Maps as constraints were used in this work, and the Viterbi algorithm was used to generate a backtracked path. In [22], maps were also considered as constraints, and the impossible paths were eliminated when the user walks for a sufficient length. The data from the trajectory were then used to construct a Wi-Fi training set. A wider integration can be found in [23,24], where PDR, Wi-Fi and map information were combined to achieve pedestrian tracking in indoor environments. Particle filter-based approaches were used to match the maps in these approaches.

Like indoor maps, landmarks, which can be detected by the unique patterns of smartphone sensor data, can be used to correct the PDR trajectory [10]. Indoor landmarks detected by HAR provide a new opportunity for indoor localization [15]. Activities, such as going upstairs (or downstairs), turning or opening doors, can be treated as landmarks. In [25], an accelerometer was used to recognize standing, walking, stairs, elevators and escalators, achieving accurate recognition. In [15], Wi-Fi, PDR and landmarks were combined to provide a highly accurate localization system. In this work, a Kalman filter was used to combine the Wi-Fi- and PDR-based localization techniques with landmarks. Without relying on Wi-Fi infrastructure, the built-in accelerometer and magnetometer in a smartphone were used to record pedestrians’ walking patterns in [26], which were then matched to an indoor map.

Semantic information has also received attention from researchers. An integrated navigation system that considers both geometrics and semantics was presented in [27]. This work proposed a semantic model that can be used to describe indoor navigation paths. Similarly, a semantic description model derived from a spatial ontology was used to describe the basic elements of navigation paths in [28]. A human-centered semantic indoor navigation system was also proposed in [29]. In order to provide services involving human factors, the system used ontology-based knowledge representation and reasoning technologies.

Although many works have been done in this field, achieving an accurate and semantic-rich trajectory in mobile environments is still a challenging task [10,11]. To improve the accuracy of trajectory locations, the user’s motion information and the indoor map were also exploited to reduce the localization errors, such as in [30]. In their work, a sequence of navigation-related action were extracted from sensor data, and an HMM was used to match the user’s trajectory with the indoor map. Inspired by this idea, the semantic acquisition and utilization were further improved in the localization process in this work. The rich location-based semantic information was extracted based on the user’s activity recognition with HAR, and a semantic model was described and constructed for indoor navigation. The semantic model can be used to not only describe the user’s location, but also to improve the user’s localization efficiency. The simultaneous localization and semantic acquisition can be considered as a significant contribution of the proposed method.

## 3. Methods

### 3.1. Location Estimation and Activity Recognition

In this section, PDR is first introduced to estimate the user’s location, and then, landmarks are applied to correct the location. Next, with the help of multiple phone sensors, HAR is used to identify the user’s activity. Finally, an HMM is proposed in order to estimate the user’s initial position.

#### 3.1.1. Landmark-Based PDR

The smartphone-based PDR system uses the inbuilt inertial and orientation sensors to track the user’s trajectory [31]. The main processes include step detection, step length estimation, direction estimation [20] and trajectory correction.

(a) Step detection:

Peak step detection [32], or the zero-crossing step detection algorithm [33], is the most frequently-used method to detect the user’s steps. To improve the robustness of the detection result, as in [34,35], the synthetic acceleration magnitude of a three-axis accelerometer is used. This is calculated as follows:

(1) $$a\left(t\right)=\sqrt{a_{x}^{2}\left(t\right)+a_{y}^{2}\left(t\right)+a_{z}^{2}\left(t\right)}-g,$$

where a(t) is the synthetic acceleration reading at time t, and the constant component g represents the Earth’s gravity.

**Figure 2:**
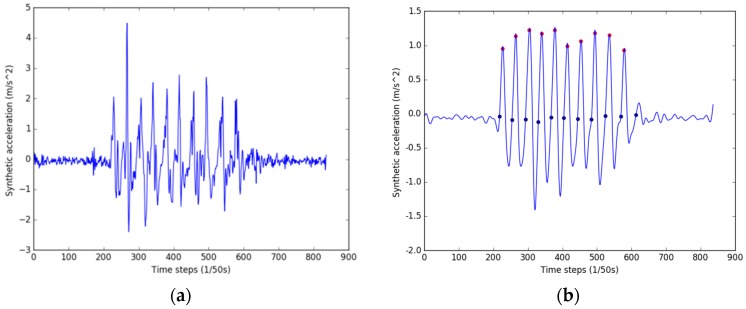
Step detection. (**a**) Raw synthetic acceleration data; (**b**) filtered data and the step detection result.

A low-pass filter is applied to smooth the data and to remove the spurious peaks, as shown in Figure 2. The step detection process is conducted according to the following conditions [10,35]:

- a(t) is the local maximum and is larger than a given threshold $\delta_{\mathrm{thr}}$.
- The time between two consecutive detected peaks is greater than the minimum step period $t_{\min}$.
- According to human walking posture, the start of a step is the zero-crossing point before the peak.

Figure 2b shows the detected peaks marked with red circles, and the blue circles represent the start and end points of the steps.

(b) Step length estimation:

The length of a step depends on the physical features of the pedestrian (height, weight, age, health status, etc.) and the current state (walking speed and step frequency) [35]. Although step length varies from step to step, even in the same person, step length can be estimated by its corresponding acceleration. A nonlinear model [34] is used to effectively estimate step length.

(2) $$l_{k}=\mu \sqrt[{4}]{a_{max}\left(k\right)-a_{min}\left(k\right)},\;0<k\leq \mathrm{num}\left(\mathrm{steps}\right)$$

where $a_{max}\left(k\right)$ and $a_{min}\left(k\right)$ are the maximum and minimum values of the synthetic acceleration during step *k*. The coefficient μ is the stride length parameter, and it can be corrected by the landmarks.

(c) Direction estimation:

Direction estimation is a challenging problem for PDR using a smartphone. The gyroscope and magnetometer in the smartphone are normally used to estimate the pedestrian’s walking direction [10]. The gyroscope and magnetometer obtain the steps’ direction during walking. An external environment can easily affect the magnetometer, which may lead to short-term heading estimation errors. Magnetic fields do not affect gyroscopes; however, gyroscopes do accumulate drift error over time [25]. In order to resolve each sensor’s drawbacks, both sensors are combined to enhance the direction estimation [13,34].

(3) $$\theta_{k}=\omega^{mag}\theta_{k}^{mag}+\omega^{gyro}\theta_{k}^{gyro},\;0<k\leq \mathrm{num}\left(\mathrm{steps}\right),$$

where $\omega^{mag}$ and $\omega^{gyro}$ are the weighting parameters on the magnetometer’s estimated direction and the gyroscope’s estimated angle, respectively. The weight value changes according to the magnitude and correlation of the gyroscope and magnetometer.

**Figure 3:**
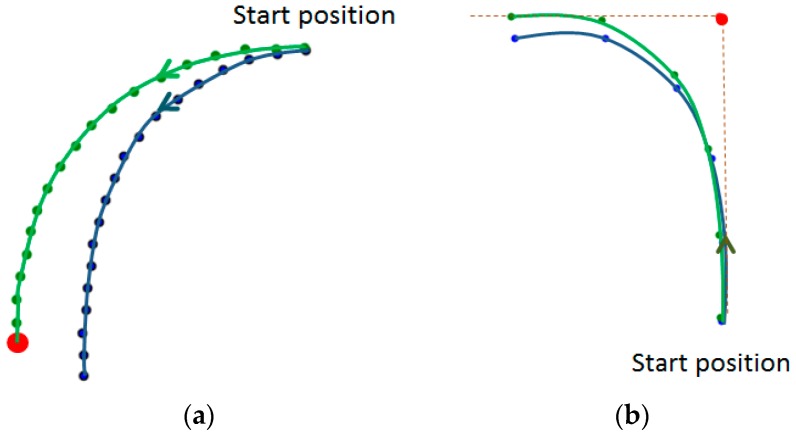
Landmark corrections. (**a**) Go straight through a landmark; (**b**) Passing a landmark when the user turns.

(d) Trajectory correction:

The raw pedestrian trajectory obtained through the above methods may encounter some bias because of the accumulated error of the PDR. To solve this problem, landmarks are used to recalibrate the errors. Depending on the location and angle of the landmarks, the step length and the angle in the raw trajectory are scaled to form a new corrected trajectory. In the process of a user’s indoor walking, two situations occur when passing a landmark. One is when a user goes straight through a landmark, as shown in Figure 3a. The other is when a user turns (see Figure 3b). As shown in Figure 3, the blue lines are the raw PDR trajectory, the green lines are the corrected trajectory and the red dots indicate the landmarks.

#### 3.1.2. Multiple Sensor-Assisted HAR

As in PDR, the synthetic three-axis accelerometer data are used as the base data in HAR. In addition, the smartphone’s magnetometer and barometer provide information about direction and height, respectively, which helps to improve the classification accuracy.

(a) Segmentation:

Three different windowing techniques have been used to divide the sensor data into smaller data segments: sliding windows, event-defined windows and activity-defined windows [36,37]. Since some specific events, such as the start and the end of a step length, are critical for pedestrian location estimation, the event-defined window approach is applied in our work. To use this approach, each step’s start and end points are detected, and then, the samples between them are regarded as a window. If no steps are detected over a period of time, the sliding window approach is used, in which two-second-long time windows with 50% overlap are selected [26,38].

(b) Feature extraction:

Two main types of data features are extracted from each time window. Time-domain features include the mean, max, min, standard deviation, variance and signal-magnitude area (SMA). Frequency-domain features include energy, entropy and time between peaks [8]. Two time-domain features are selected—the mean and standard deviation—because they are computationally inexpensive and sufficient to classify the activities.

(c) Classification:

A supervised learning method is adopted to infer user activities from the sensory data [7]. A number of different classification algorithms can be applied in HAR, such as decision tree (DT), *k*-nearest neighbor (KNN), support vector machine (SVM) and naive Bayes (NB) [8,9,38]. Due to the simplification and high accuracy of KNN, the KNN algorithm (see Algorithm 1) is selected to classify four activities: standing, going up (or down) stairs, walking and opening a door.

**Algorithm 1.** KNN.Input:Samples that need to be categorized: $X_{j}$; the known sample pairs: ($X_{i}$, $y_{i}$)Output:Prediction classification: $y_{j}$
1:**for** every sample in the dataset to be predicted **do**2: calculate the distance between ($X_{i}$, $y_{i}$) and the current sample $X_{j}$
3: sort the distances in increasing order4: select the k samples with the smallest distances to $X_{j}$
5: find the majority class of the k samples6: return the majority class as the prediction classification $y_{j}$
7:**end For**

**Figure 4:**
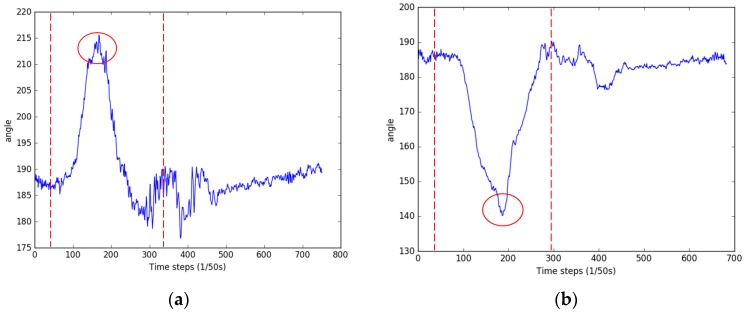
The magnetometer changes when a door is opened. (**a**) Opening a south-facing door, where the door handle is to the right; (**b**) opening a south-facing door, where the door handle is to the left.

In order to improve the classification accuracy of indoor activities, a barometer is used to determine upstairs or downstairs and to locate the user’s floor. A magnetometer is used to assist in identifying the door-opening activity. Different ways of opening the door correspond to different magnetometer reactions. However, their similar performance patterns can be extracted by detecting the peak value change of the magnetometer in the sliding window. As shown in Figure 4, when the user opens a door, the magnetometer readings change significantly within a short time and then quickly return to the previous readings. Thus, the door-opening activities can be effectively identified.

#### 3.1.3. The Hidden Markov Model 

When the user’s initial location is unknown, HMM is used to match the motion sequence with indoor landmarks. PDR and HAR also provide useful information for matching and location estimation. As a widely-applied statistical model, HMM has a unique advantage in processing natural language, and it can capture the hidden states in a sequence of motion observations [30,36]. There are five basic elements in HMM: two sets of states (N, M) and three probability matrices (A, B, π).

Because of the unique indoor environment, HMM is presented as follows:

(1) N represents the hidden states in the model, which can be transferred between each other. The hidden states in HMM are landmark nodes in the indoor environment, such as a door, stairs or a turning point.
(2) M indicates the observations of each hidden state, which are the user’s direction selection (east, south, west and north) and the activity result from HAR.
(3) A and B state the transition probability and the emission probability, respectively. The pedestrian moves indoors from one node to another, and when the direction of the current state is determined, the reachable nodes are reduced. In order to reduce the algorithm’s complexity, A and B are combined to give a transition probability set C. [$C_{e}$, $C_{s}$, $C_{w}$, $C_{n}$] represent the transition probabilities of different directions.
(4) π is the distribution in the initial state. The magnetometer and barometer provide direction and altitude information when the user starts recording, which helps to reduce the number of candidate nodes in the initial environment. If the starting point is unknown, the same initial probability is given.

The Viterbi algorithm uses a recursive approach to find the most probable sequence of hidden states. It calculates the most probable path to a middle state, which achieves the maximum probability in the local trajectory. Choosing the state’s maximum local probability can determine the best global trajectory. However, in the indoor environment, using a partial maximum probability to obtain the global path is not appropriate, because the probability between hidden states could be zero, and a local best trajectory could become a dead trajectory in the next moment. In this study, the distance information from PDR and the activities information from HAR are combined with the Viterbi algorithm to compute the most likely trajectory. The improved Viterbi algorithm (Algorithm 2) is proposed as follows:

**Algorithm 2.** Improved Viterbi algorithm.Input:The proposed HMM tuples $<N=\left\{n_{i}|i=1,2,\ldots ,N_{N}\right\},$
$M=\left\{m_{i}|i=1,2,\ldots ,N_{M}\right\},\;C,\;\pi >$; HAR classification results $H=\{h_{i}|i=1,2,\ldots ,N_{H}\}$; PDR distance information $D=\{d_{i}|i=1,2,\ldots ,N_{D}\}$; Initial direction of magnetometer O; Initial pressure of barometer F; $d_{\sigma }$ is the distance threshold.Output:Prediction trajectory.1:$O_{start}\leftarrow O$, $F_{start}\leftarrow F$/* Determine the initial orientation and floor2:**for**
i from 1 to $N_{M}$
**do**3: **for** each path pass through $n_{i-1}$ to $n_{i}$
**do**4:  **if** ((Distance($d(n_{i-1}$), $d(n_{i}$)) - $d_{i}$)<$d_{\sigma }$) and ($P\left(n_{i}\right)$>0) **then** /* Determine whether the distance between two landmark nodes coincides with the distance information estimated by PDR5:   $\mathrm{Path}\left(N_{s},\;P\left(N_{s}\right)\right)\leftarrow$Obtain the subset data6:  **end if**7: **end for**8:**end for**9:**for**
${\mathrm{path}}_{j}$ in $\mathrm{Path}\left(N_{s},\;P\left(N_{s}\right)\right)$
**do**10: $H\left({\mathrm{path}}_{j}\right)=\{h_{i}|i=1,2,\ldots ,N_{H}\}\leftarrow$Obtain the landmark data set11: **if**
$H\left({\mathrm{path}}_{j}\right)$ match with HAR data H
**then**12:  $\mathrm{Path}\left(N_{f},\;P\left(N_{f}\right)\right)\leftarrow$Add this trajectory to the final trajectory data set 13:**end for**14return Max($\mathrm{Path}(N_{f},\;P\left(N_{f}\right))$)/* Return the trajectory of the maximum probability

With the determination of the user’s trajectory, the initial position can be obtained through the first landmark point and the PDR information.

### 3.2. Semantic Landmark Model

#### 3.2.1. Trajectory Information Collection

**Figure 5:**
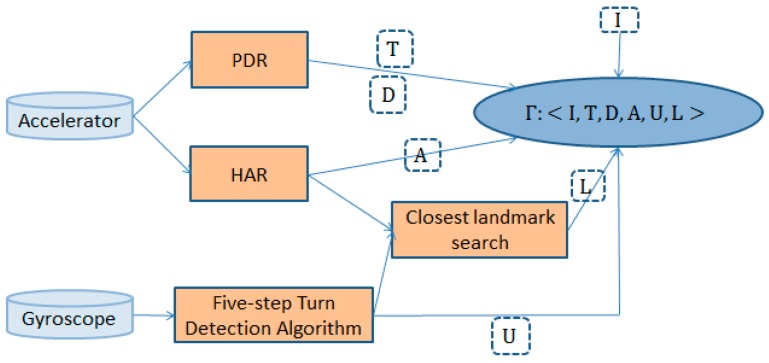
The trajectory information collection process.

**Definition 1.** *Trajectory information: A trajectory is defined as a six-tuple* Γ:〈I, T, D, A, U,L〉, *where I is the ID of the trajectory, and* T *and* D *are the timestamp and position information, respectively, of each step.* U *is the direction change list; *A *is the activities information list; and L is the landmark list. Figure 5 shows the trajectory information collection process*.

For example, if a user went from Entrance (ET) to Room 108, I is assigned ET–R108.

From PDR, the timestamp and xyz coordinate value for each step can be obtained and can be represented as:

(4) $$T=\left[t_{1},t_{2}\ldots \;t_{n}\right],$$

(5) $$D=\left[\left(x_{1},y_{1},z_{1}\right),\;\left(x_{2},y_{2},z_{2}\right),\ldots ,\;\left(x_{n},y_{n},z_{n}\right)\right],$$

where n denotes the number of steps detected.

Using the HAR method, the user’s activity information can be collected. Hence, A can be given as follows:

(6) A= {Standing, Walking, Going up stairs, Opening a door}

The direction change list U can be obtained by the gyroscope. It should be noted that we detected only large directional changes (>15°), and thus, walking along a smaller arc was not detected. Because most of the turns could be completed in less than five steps, a five-step turn detection method (see Algorithm 3) is proposed to determine the direction change activity.

**Algorithm 3.** Five-step turn detection algorithm.Input:Angle value sequence θ = [$\theta_{1},\theta_{2},\ldots \theta_{n}]$Output:Direction change list U.1:$\theta_{\max}\leftarrow$Findpeaks(θ)/* Find the local maximum sequence2:**for**
$\theta_{i}$ in $\theta_{\max}$
**do**3: **if** ($\theta_{i}>15$ or $\theta_{i}<-15$) and (i>2) **then**4:  $\theta_{\mathrm{sum}}$ = $\mathrm{sum}\left[\theta_{i-2}:\theta_{i+2}\right]$5: **if** ($\theta_{\mathrm{sum}}>30\;and\;\theta_{\mathrm{sum}}\leq 60$) **then**6:  U.add(“Go left”)7: **else if** ($\theta_{\mathrm{sum}}>60\;and\;\theta_{\mathrm{sum}}\leq 120$) **then**8:  U.add(“Turn left”)9: **else if** ($\theta_{\mathrm{sum}}>-60\;and\;\theta_{\mathrm{sum}}\leq -30$) **then**10:  U.add(“Go right”)11: **else if** ($\theta_{\mathrm{sum}}>-120\;and\;\theta_{\mathrm{sum}}\leq -60$) **then**12:  U.add(“Turn right”)13: **else if** ($\theta_{\mathrm{sum}}>120\;or\;\theta_{\mathrm{sum}}\leq -120$) **then**14  U.add(“Turn around”)15:**end for**16:return U


For example, U can be described as follows:

(7) U={Go left, Turn left,Turn right}

Since the landmarks are used as the key points in a trajectory, a landmark list can be used to denote a trajectory. According to the time series, a landmark list L is detected in a trajectory. Three types of landmarks (stairs, turns and doors) are added to list L according to the following rules:

- If the going-up-(or -down)-stairs activity is detected, the nearest stairs landmark is added to L.
- If direction change activity (see Algorithm 3) is detected, the nearest turn landmark is added to L.
- If a door-opening activity is detected, the nearest door landmark is added to L.

#### 3.2.2. Semantics Extraction

**Figure 6:**
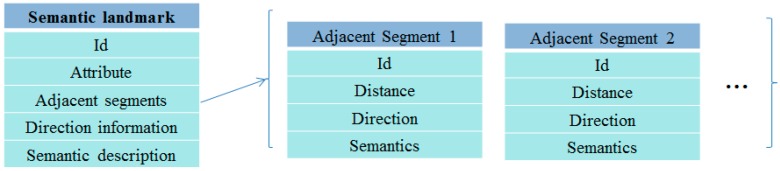
Semantic landmark and adjacent segments. An adjacent segment consists of four parts: Id, distance, direction and semantic description. Id is the identifier of a segment. Distance represents the distance between the two landmarks that make up the segment. Direction represents the direction of the segment. Semantics indicates the semantic information that can be obtained.

**Definition 2.** *Semantic landmark: A semantic landmark* S[l] *consists of five parts: Id, attribute, adjacent segments, direction information and semantic description. Id is the landmark identifier. Attribute is one of the three types of landmarks: stairs, turn, or door. Adjacent segments contain the distance and semantic information between the current landmark and the next landmarks. Direction information and semantic description indicate the direction information and the semantic information when the user passes the landmark, respectively, as shown in Figure 6*.

A sequence of landmarks can denote a trajectory. Therefore, adding semantic information to the landmarks and their adjacent segments can describe the trajectory. The semantic description of trajectories is expressed as follows:

(8) $$S\left[t\right]=\left\{S\left[seg_{s-1}\right],\;S\left[l_{1}\right],S\left[seg_{1-2}\right],S\left[l_{2}\right],\;\ldots \;S\left[l_{n}\right],\;S\left[seg_{n-e}\right]\right\},$$

where S[t] indicates the semantics of trajectory t, and n is the number of landmarks for this trajectory. $S\left[l_{n}\right]$ represents the semantics of landmark $l_{n}$. $S\left[seg_{s-1}\right]$ denotes the semantics of the region from the start point to the first landmark point. Similarly, $S\left[seg_{n-e}\right]$ denotes the semantics of the region from the last landmark point to the end.

A semantic landmark or an adjacent segment can store multiple semantics and provides semantics based on the detected activity. According to the trajectory information Γ:〈I, T, D, A, U,L〉, the semantic information can be obtained as shown in Table 1.

Detected (U) and Find (L(turn)) indicate that turn activity is detected and that a nearby turn landmark is found. If the user’s activity information is detected, but there are no corresponding landmarks nearby, this activity’s semantics is added to the corresponding adjacent segment, as shown in Table 2.

**Figure 7:**
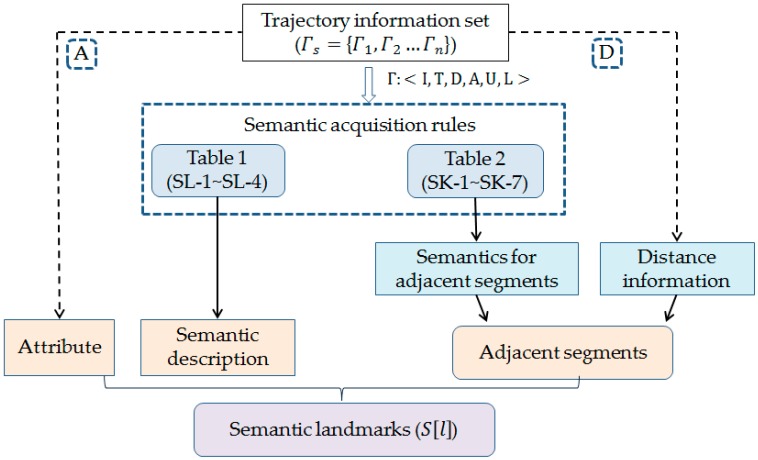
Semantic landmark construction process.

According to the semantics acquisition rules shown in Table 1 and Table 2, the semantic landmarks are constructed as shown in Figure 7.

As the above process shows, both semantic information and distance information are added to the semantic model. The order information (e.g., “Turn left at the 2nd turning point”) is added according to the following rule:

- If the current landmark’s adjacent segments contain multiple turn or door landmarks and they have the same semantics, sort them by distance and then provide them with the order information ${\mathrm{Order}}_{\mathrm{turn}}$ or ${\mathrm{Order}}_{\mathrm{door}}$.

Set S denotes the semantics obtained, such as {S1: [‘Go left’], S2: [‘Go up the steps’], S3: [‘Turn left’]}. If the order information is obtained simultaneously, it can be expressed as {S_order: [‘at ${\mathrm{Order}}_{\mathrm{turn}}$ turn point’] or [‘at ${\mathrm{Order}}_{\mathrm{door}}$ door’]}.

## 4. Experiment

**Figure 8:**
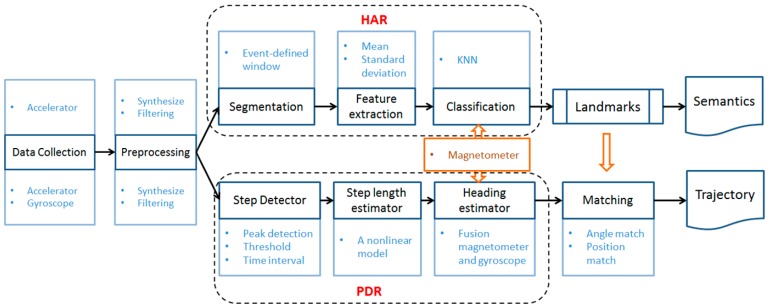
The experiment’s overall process.

The experiment was performed at the State Key Laboratory of Information Engineering in Surveying, Mapping and Remote Sensing (LIESMARS) at Wuhan University, China. An Android mobile phone and the indoor floor plans of LIESMARS were used in the experiment. It should be noted that, in this study, only the hand-held situation was considered. The experimental process is shown in Figure 8.

In the following, the pre-knowledge is first provided. Multiple user trajectories are then presented, including a trajectory on a single floor, a trajectory on multiple floors and a trajectory without knowing the starting point. Finally, the semantics acquisition process and results are described.

### 4.1. Pre-Knowledge

**Figure 9:**
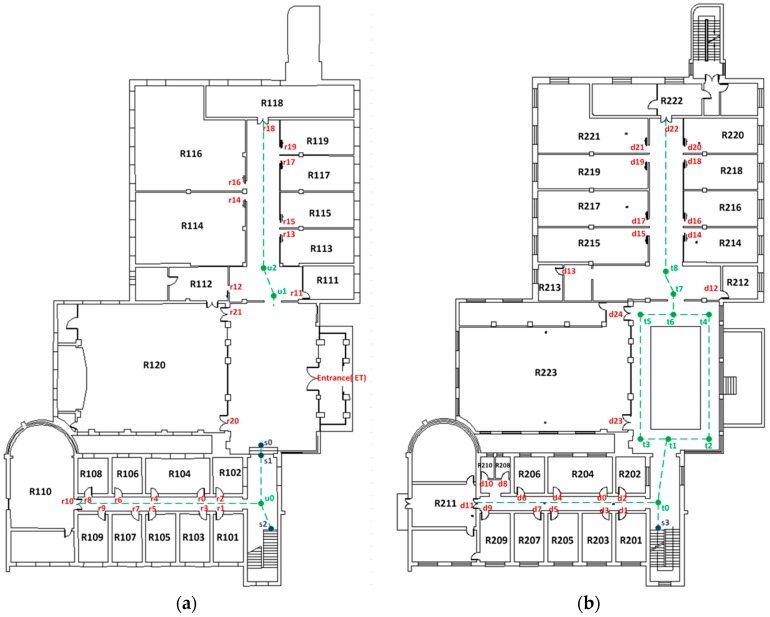
Landmarks. (**a**) Landmarks of the first floor; (**b**) landmarks of the second floor.

Door points, stair points and turn points were used as landmarks in the experiment, as shown in Figure 9. In indoor spaces, pedestrians tend to walk along a central line, and they tend to go in a straight line between places. The intersection of the corridor area’s centerline was therefore selected as a landmark. Most turn points near door points were not assumed to be landmarks, because the door points could replace them as landmarks. The above principles were used to generate the landmarks. The proposed approach does not focus on landmark extraction, but on trajectory generation and the steps in the semantics acquisition.

The smartphone’s barometer and magnetometer were used to determine the initial orientation and locate the user’s floor, respectively. However, they need to be analyzed first. We collected barometer data at eight different locations on each layer, as shown in Table 3. As the change in barometer readings in the same layer was not significant, we used the average of the collected barometer readings as a benchmark to determine the user’s floor. If the current barometer reading is within ±0.1 hpa of B(f1) or B(f2), the corresponding floor is determined.

**Figure 10:**
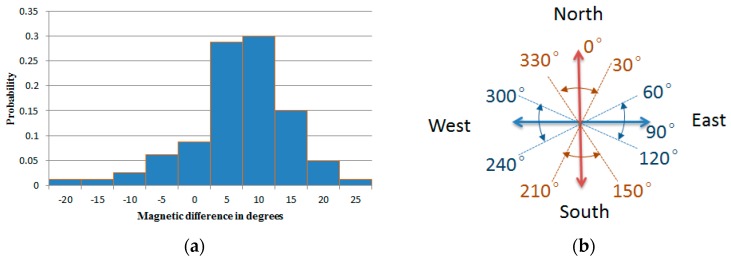
The direction information obtained by the magnetometer. (**a**) Distribution of the magnetic differences; (**b**) direction information.

Compared to the barometer, the magnetometer is more unstable, so 80 north-facing magnetometer readings were collected at various locations within the building. The distribution of the difference between the collected magnetometer data and True North is shown in Figure 10a. Most of the magnetic differences are between −5° and 15° and occasionally more than 20°. Based on the above data, a threshold of 30° was chosen to determine the direction semantics of the initial position. Direction information like ‘north’ (330 < θ < 360, 0 ≤ θ < 30), ‘east’ (60 ≤ θ <120), ‘south’ (150 ≤ θ <210) and ‘west’ (240 ≤ θ < 300) can be obtained (Figure 10b) when the magnetometer reading θ of the user’s initial position is acquired.

In addition, the user’s step length parameters need to be determined over a short distance (0.45 in our experiment). When the user goes upstairs, the horizontal and vertical distances of each step are given as fixed values (0.3 m and 0.15 m in the experiment).

### 4.2. Trajectory Generation and Correction

To present a user’s trajectory, HAR is performed to identify landmarks, and then, these landmarks are used to correct the PDR trajectory.

**Figure 11:**
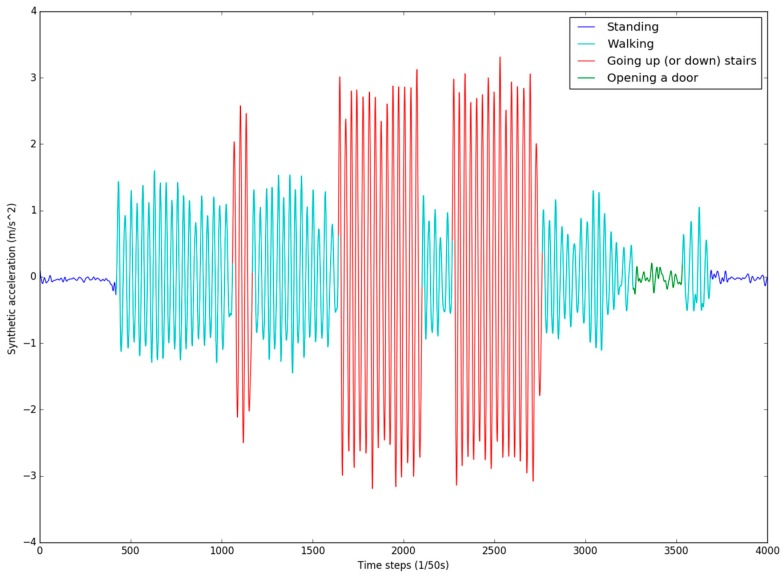
The activity sample collection of trajectory Entrance (ET)–R201.

Because the HAR training set requires a variety of activities, 25 trajectories from the entrance to each room on the second floor were chosen. If a room has multiple doors, each door corresponds to a trajectory. In order to obtain information from standing activity samples, the user needs to stand for a while at the beginning point and the end point of each trajectory. A training sample is shown in Figure 11.

**Figure 12:**
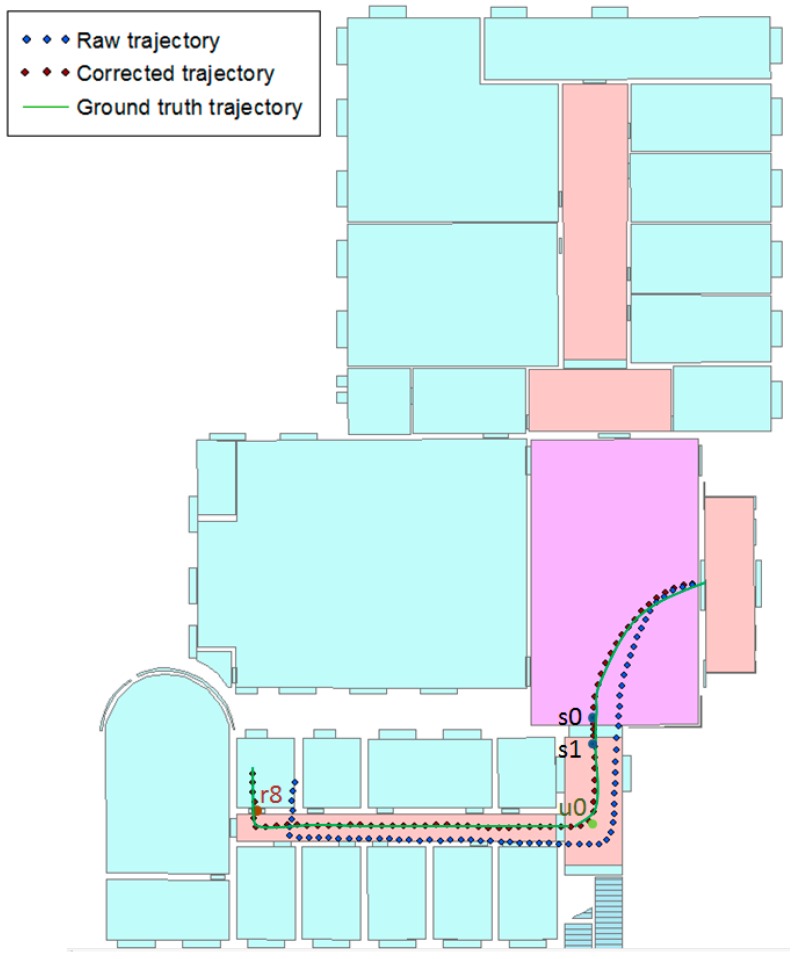
The trajectory of ET–R108. The raw trajectory is without landmarks, and the corrected trajectory is with landmarks.

Landmarks that the user passes can be determined using the activity classification results provided by HAR, and then, the user’s trajectory can be corrected. Figure 12 shows the trajectory from the entrance to Room 108. The blue points indicate the raw trajectory, and the red points indicate the corrected trajectory. The corrected trajectory is extremely close to the ground truth trajectory. In addition, only four landmarks were used: stairs landmarks s0 and s1, turn landmark u0 and door landmark r8.

**Figure 13:**
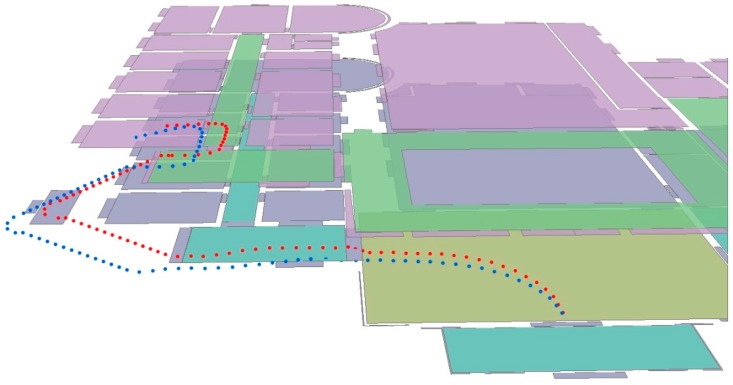
The trajectories on multiple floors.

For trajectories on multiple floors, height information is added to each step point. Figure 13 shows the trajectory from the entrance to Room 201. The blue points and red points indicate the raw trajectory and the corrected trajectory, respectively.

When the user’s initial location is unknown, the direction observation sequence is obtained from the direction sensors. Information about position and activities is obtained from PDR and HAR, respectively. When the user trajectory is determined, the starting position can be inferred from PDR.

**Figure 14:**
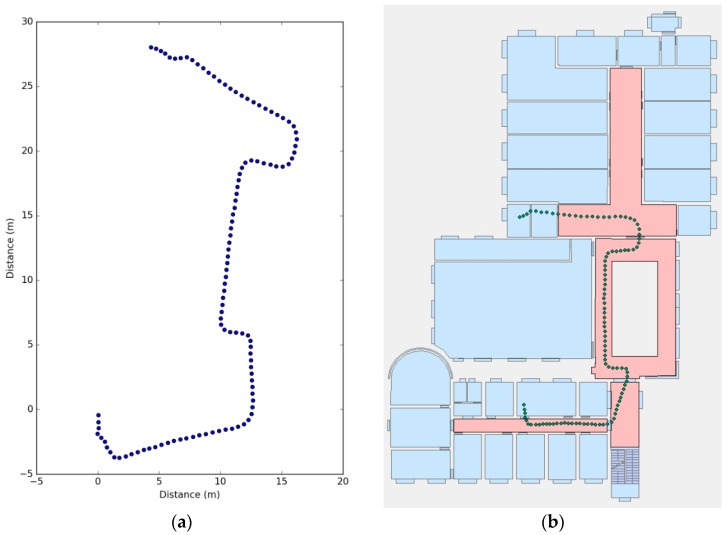
Trajectory matching results. (**a**) Raw PDR trajectory; (**b**) matching trajectory.

The user went from point S to point E. The trajectory when using only PDR is shown in Figure 14a, and the direction observation sequence is obtained.

(9) M={south, east, north, west, north, east, north, west}

From PDR, the distance between the landmarks of two adjacent observation sequences is obtained, which is denoted by D. For example, $D_{s}$ represents the distance from the starting point to the first landmark, and $D_{1-2}$ denotes the distance from the first landmark to the second landmark. $D_{e}$ represents the distance from the end point to the last landmark.

(10) $$D=\left\{D_{s},\;D_{1-2},\;D_{2-3},\ldots ,\;D_{e}\right\}$$

Information about activities is obtained from HAR.

(11) A={Standing, Opening a door, Walking, Opening a door, Walking, Standing}

The matching process used in the algorithm is shown in Table 4. To simplify the proposed model, a flag was abstracted to express similar landmarks. For example, DN stands for the adjacent doors at the north side of the corridor: dn=[d0,d2,d4,d6,d8,d10], ds=[d1,d3,d5,d7,d9], dw = [d15,d17,d19,d21], de= [d14,d16,d18,d20] (see Figure 9). The virtual landmark E indicates the connecting points between the doors and the corridor. For example, E(d3) indicates the connecting point between door d3 and the corridor.

A trajectory is represented by a list, and the elements in the list represent the points that have been passed. The HAR results are denoted by A. $A_{s}$ = (s, w, o) indicates the sequence of activities from the start point to the first landmark, which is “Standing-Walking-Opening a door”.

It should be noted that we used the real landmark coordinates to correct the PDR results when landmarks were detected. As the trajectory ended, d_e_ was given to estimate the final position, and the matching trajectory was obtained.

### 4.3. Semantics Extraction

According to the proposed semantics extraction method described in Section 3.2, the semantics for landmarks in trajectory ET–R108 (in Figure 12) were obtained as shown in Table 5. The start and end points were considered as virtual landmarks, which have no specific attributes or fixed locations.

After sufficient trajectories were acquired, the landmarks’ full semantics could be obtained. Taking the turn landmark (u0) as an example, the complete semantics are as shown in Table 6. In addition, the order of the landmarks could be obtained using the method described in Section 3.2.2.

According to the above tables and our semantics model, the three trajectories presented in Section 4.2 can be described as follows:

ET–R108: {[‘Go left’], [‘Go up the steps’], [‘Turn right’], [‘Go straight’,’ Turn right (at 5th door)’], [‘Go into the door’]}

ET–R201: {[‘Go left’], [‘Go up the steps’], [‘Go straight’, ‘Go upstairs’], [‘Turn left], [‘Turn left (at 1st door)’], [‘Go into the door’]}

R204–R213: {[‘Go out the door’], [‘Turn left’], [‘Go straight’], [‘Turn left’], [‘Turn left’], [‘Turn right’], [‘Go straight’], [‘Turn right’], [‘Turn left’], [‘Turn left’], [‘Go straight’], [‘Go into the door’]}

The construction of complete semantics for all of the indoor landmarks requires a large amount of trajectory data. However, the proposed approach only considers the complete semantics of key landmarks and the partial semantics of non-key landmarks, because they can describe most of the user’s activities.

## 5. Discussion

Firstly, the performance of the HAR classification is evaluated. The location errors in a trajectory are then described. Finally, the accuracy of the landmark matching is analyzed.

### 5.1. Error Analysis

#### 5.1.1. HAR Classification Error

In order to evaluate the performance of the HAR classification, 10-fold cross-validation [39] was used. In this method, the dataset is divided into 10 parts: nine parts are used for training, and one part is used for testing each iteration. The classification accuracy of the common classifiers was compared with the proposed classifier, and two different window segmentation approaches were compared. Since the error rate of our step detection is quite low, at 0.19% (total steps: 3092; error detection steps: six), it is more convenient to use the event-defined window approach to sense the user’s activity. As shown in Table 7, the results show that the event-defined window approach performs better than the sliding window approach, which applies two-second-long time windows with a 50% overlap.

Many different performance metrics could have been be used to evaluate the HAR classification [8]. A confusion matrix was adopted, which is a method commonly used to identify error types (false positives and negatives) [40]. Several different performance metrics—accuracy (the standard metric to express classification performance), precision, recall and F-measure—could be calculated based on the matrix [9]. Table 8 shows the confusion matrix used to evaluate the results of the KNN classification activities.

As shown in Table 8, the proposed method achieves an extremely high accuracy (>99%) in detecting stairs and walking activities. Some errors occur in identifying door-opening and standing activities. However, by detecting the magnetometer change, it is easy to distinguish between these two activities, thereby reducing the amount of errors.

#### 5.1.2. Localization Error

**Figure 15:**
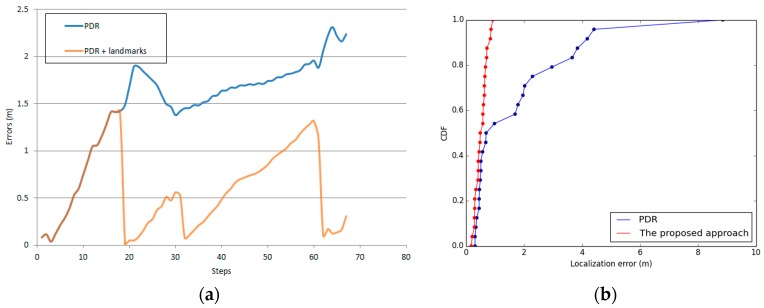
Localization error. (**a**) Localization error of trajectory ET–R108; (**b**) the cumulative error distribution of the 25 test trajectories.

The localization error of trajectory ET–R108 is shown in Figure 15a; the blue line indicates the original PDR trajectory, and the orange line indicates the location errors after only the landmarks were corrected. The results show that the PDR errors increase with distance, and a high average localization accuracy (0.59 m) is achieved when we use the landmarks to correct the cumulative errors. Figure 15b shows the cumulative error distribution of the 25 test trajectories. We can see that the proposed approach is more stable than using only PDR, and the average error is reduced from 1.79 m to 0.52 m.

#### 5.1.3. Landmark Matching Errors

**Figure 16:**
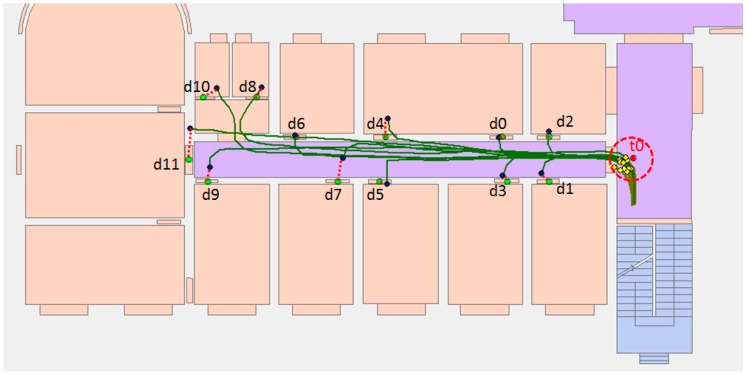
Landmark matching. The red point is the turn landmark, and the green points are the door landmarks. The yellow points are the PDR position when a turn is detected. The blue points are the PDR position when opening a door is detected. The dashed red line indicates the nearest landmark points.

The shortest distance method was used to match the landmarks. As shown in Figure 16, the result matches the partial trajectories.

Although the trajectories were corrected at the turn landmark (the red point), the PDR-estimated user location still introduced errors, particularly when the user was far from the previous landmark. In the experiment, an error occurred because the distances of d18 and d20 were extremely close and far from the turning point landmark (t6). We can also see that a similar error occurred in turn landmark t5, which is matched to landmark t6 (see Table 9).

### 5.2. Comprehensive Comparison

**Figure 17:**
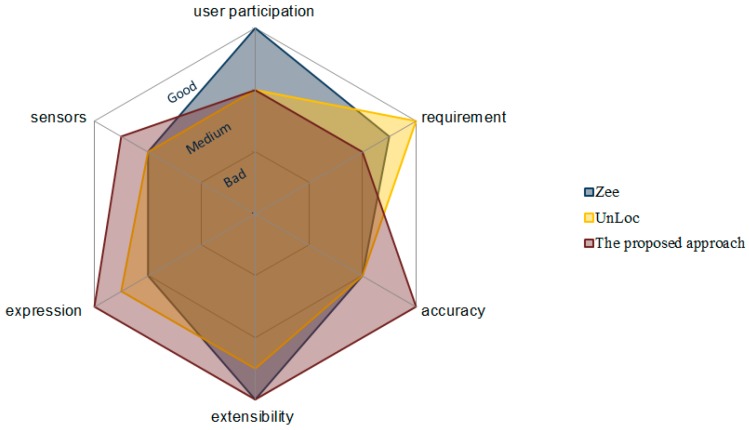
Overall score of Zee, UnLoc and the proposed approach.

Some similar indoor localization schemes, which require no additional devices or expensive labor, are compared in terms of requirement, sensors, user participation, accuracy, expression and extensibility in Table 10. Each technique has its own advantages. Zee [22] tracked a pedestrian’s trajectory without user participation, and a Wi-Fi training set was simultaneously collected, which can be used in Wi-Fi fingerprinting-based localization techniques. UnLoc [25] only needs a door location as the basic input information and simultaneously computes the user’s location and detects various landmarks. Compared to the above localization schemes, the proposed approach needs more basic information; however, the information allows us to obtain a better localization accuracy. Moreover, a semantic landmark model was constructed during the localization process, which can be used not only to describe the user’s trajectory, but also to improve the localization efficiency. The overall scores of the three approaches are shown in Figure 17.

### 5.3. Computational Complexity

**Figure 18:**
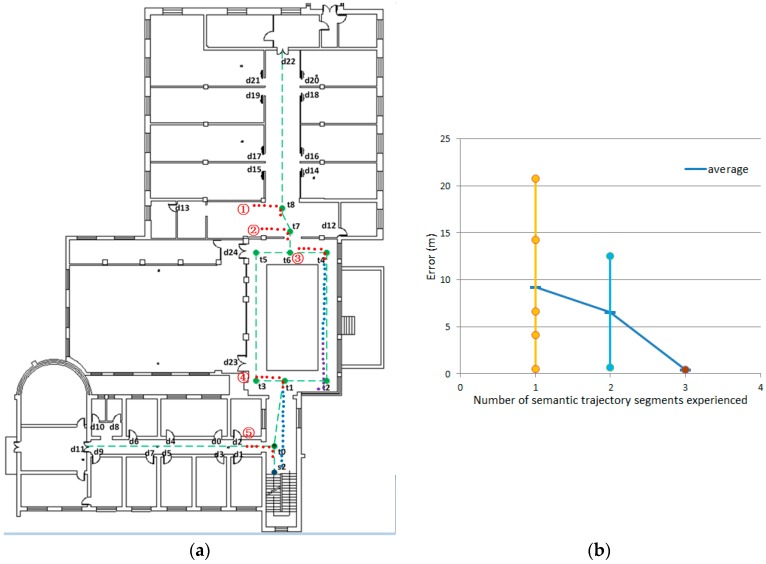
Semantic matching of trajectories. (**a**) The trajectories after semantic matching; (**b**) the trajectory error.

In order to verify the validity of the semantic model for localization and better analyze the computational complexity of semantics-assisted localization method, a further experiment was conducted (Figure 18a). In this experiment, the user started from any place on the second floor, and we wanted to determine the user’s trajectory as soon as possible. The overall error and time complexity in the trajectory matching process are used to evaluate the proposed method.

As shown in Figure 18b, although only a few semantics are provided, the trajectory error drops rapidly (the average error drops from 9.25 m to 0.48 m). When the first semantic information is obtained from the trajectory, there are five trajectories satisfying the condition. In order to match the semantic information, each landmark point needs to be traversed once, so the time complexity is O(N). The next search only needs to traverse the semantics of the trajectory segments that satisfy the previous condition. These trajectory segments are ‘$l_{t8}l_{t7}$’, ‘$l_{t8}l_{t6}$’, ‘$l_{t7}l_{t6}$’, ‘$l_{t4}l_{t2}$’, ‘$l_{t1}l_{t0}$’, ‘$l_{t1}l_{s2}$’, ‘$l_{t0}l_{s2}$’, and the time complexity is O(7). The trajectory is determined after matching the third semantic information, and the trajectory error is similar to the previous trajectory localization experiment, where the initial location was known. The above description is provided in Table 11. Compared to the traditional trajectory matching method, which yields a time complexity of O(NT) or O(N2T), the proposed semantic matching method is more efficient. Since it does not need to traverse all of the states every time, the time complexity is much less than O(NT).

## 6. Conclusions

In this paper, PDR, HAR and landmarks have been combined to achieve indoor mobile localization. The landmark information was extracted from indoor maps, and then, HAR was used to detect the landmarks. These landmarks were then used to correct the PDR trajectories and to achieve a high level of accuracy. Without knowing the initial position, HMM was performed to match the motion sequence to the indoor landmarks. Because semantic information was also assigned to the landmarks, the semantic description of a trajectory was obtained, which has the potential to provide more applications and better services. Moreover, the experiment was implemented in an indoor environment, to fully evaluate the proposed approach. The results not only show a high localization accuracy, but also confirm the value of semantic information.

More extensive research can be studied in the future. For example, phone sensing could be used to recognize more activities, particularly complex activities, and more semantic information could be extracted. In addition, complex experimental conditions, such as various trajectories, a location-independent mobile phone and all kinds of users, could be included in future studies. The semantic model used in this study does not contain all possible semantics, and rich semantic information could be obtained by using the data from crowdsourced trajectories. Furthermore, real-time localization (including semantic information) is also the priority of our future work.

## Figures and Tables

**Table 1:** Semantics acquisition rules for landmarks.

Id	Conditions (C)	Semantics (S)
SL-1	A = ‘Walking’, Detected (U) and Find (L(turn))	‘Go left’, ‘Go right’, ‘Turn left’, ‘Turn right’, ‘Turn around’
SL-2	A = ‘Opening a door’, Find (L(door)) and $l_{\mathrm{pre}}=\emptyset$ and $l_{\mathrm{next}}=\emptyset$	‘Opening a door’
SL-3	A = ‘Opening a door’, Find (L(door)) and $l_{\mathrm{pre}}!=\emptyset \;\mathrm{or}\;l_{\mathrm{current}}$	‘Go into the door’
SL-4	A = ‘Opening a door’, Find (L(door)) and $l_{\mathrm{next}}!=\emptyset \;\mathrm{or}\;l_{\mathrm{current}}$	‘Go out of the door’

SL indicates the identity of the rule. A = {‘Standing’, ‘Walking’, ‘Going up (or down) stairs’, ‘Opening a door’}, U = {‘Go left’, ‘Go right’, ‘Turn left’, ‘Turn right’, ‘Turn around’}. *L* is the landmark list: L(turn), L(stairs) and L(door) are the turn, the stairs and the door landmarks. $l_{\mathrm{current}}$, $l_{\mathrm{pre}}$ and $l_{\mathrm{next}}$ represent the current landmark, the previous landmark and the next landmark.

**Table 2:** Semantics acquisition rules for landmark segments.

Id	Conditions (C)	Semantics (S)
SK-1	A = ‘Standing’, Detected (U)	‘Turn left’, ‘Turn right’, ‘Turn around’
SK-2	A = ‘Walking’, Detected (U) and Unfound (L(turn))	‘Go left’, ‘Go right’, ‘Turn left’, ‘Turn right’, ‘Turn around’
SK-3	A = ‘Walking’, Undetected (U), $D_{\mathrm{walking}}$ > $D_{\mathrm{thresold}}$	‘Go straight’
SK-4	A = ‘Going up(or down) stairs’, Find (L(stairs)) and $Z_{\mathrm{current}}$ < $Z_{\mathrm{next}}$, $T_{\mathrm{stairs}}$ < 5 s	‘Go up the steps’
SK-5	A = ‘ Going up(or down) stairs, Find (L(stairs)) and $Z_{\mathrm{current}}$ > $Z_{\mathrm{next}}$, $T_{\mathrm{stairs}}$ < 5 s	‘Go down the steps’
SK-6	A = ‘ Going up(or down) stairs, Find (L(stairs)) and $Z_{\mathrm{current}}$ < $Z_{\mathrm{next}}$, $T_{\mathrm{stairs}}$ > 5 s	‘Go upstairs’
SK-7	A = ‘ Going up(or down) stairs, Find (L(stairs)) and $Z_{\mathrm{current}}$ > $Z_{\mathrm{next}}$, $T_{\mathrm{stairs}}$ > 5 s	‘Go downstairs’

SK indicates the identity of the rule. A = {‘Standing’, ‘Walking’, ‘Going up (or down) stairs’, ‘Opening a door’}, U = {‘Go left’, ‘Go right’, ‘Turn left’, ‘Turn right’, ‘Turn around’}. *L* is the landmark list: L(turn), L(stairs) and L(door) are the turn, the stairs, and the door landmarks. The duration of ‘Standing’, ’Walking’ and ‘Going up (or down) stairs’ activities are represented by $T_{\mathrm{standing}}$, $T_{\mathrm{walking}}$ and $T_{\mathrm{stairs}}$, respectively. From *D*, we can obtain the distance and height information$;\;D_{\mathrm{walking}}$ indicates the walk distance; $Z_{\mathrm{current}}$ represents the *z* value of the current landmark; and $Z_{\mathrm{next}}$ represents the *z* value of the next landmark.

**Table 3:** Barometer readings.

Floor	1	2	3	4	5	6	7	8	Average (hpa)
f1	1020.91	1020.92	1020.92	1020.9	1020.88	1020.86	1020.85	1020.87	B(f1) = 1020.89
f2	1020.32	1020.34	1020.34	1020.33	1020.29	1020.3	1020.33	1020.32	B(f2) = 1020.32

**Table 4:** Trajectory matching results. E = east, S = south, W = west, N = north. s = standing, w = walking, u = going up stairs, d = going down stairs, o = opening a door.

Observation Sequence	Trajectories	Distance and Activities Information	Trajectories after
{‘S’, ‘E’, ‘N’}	[d4, E(d4), t0], [t5,t3, t2]	{$D_{s}$ = 1.86, $D_{1-2}$ = 1.69, $D_{2-3}$ = 10.9} {$A_{s}$ = (s, w, o), $A_{1-2}$ = (w), $A_{2-3}$ = (w)}	[d4, E(d4), t0]
{‘S’, ‘E’, N’,’W’}	[d4, E(d4), t0, t1]	$D_{3-4}$ = 6.73, $A_{3-4}$ = (w)	[d4, E(d4), t0, t1]
{‘S’, ‘E’, N’,’W’, ‘N’}	[d4, E(d4), t0, t1, t3]	$D_{4-5}$ = 2.1, $A_{4-5}$ = (w)	[d4, E(d4), t0, t1, t3]
{‘S’, ‘E’, N’,’W’, ‘N’, ‘E’}	[d4, E(d4), t0, t1, t3, t5]	$D_{5-6}$ = 13.73, $A_{5-6}$ = (w)	[d4, E(d4), t0, t1, t3,t5, t6]
{‘S’, ‘E’, N’,’W’, ‘N’, ‘E’, ‘N’}	[d4, E(d4), t0, t1, t3, t5, t6]	$D_{6-7}$ = 4.1, $A_{6-7}$ = (w)	[d4, E(d4), t0, t1, t3,t5, t6]
{‘S’, ‘E’, N’,’W’, ‘N’, ‘E’, N’,’W’}	[d4, E(d4), t0, t1, t3, t5, t6, t8],[d4, E(d4), t0, t1, t3, t5, t6,E(dw)]	$D_{7-8}$ = 4.1, $A_{7-8}$ = (w)	[d4, E(d4), t0, t1, t3,t5, t6, t8]
	[d4, E(d4), t0, t1, t3, t5, t6, t8]	$D_{8-9}$ = 10.94, $D_{e}$ = 2.8 $A_{8-9}$ = (w, o, w)	[d4, E(d4), t0, t1, t3,t5, t6, t8, d13]

**Table 5:** Landmark semantics.

Landmark	Name	Expression
Entrance	Id	ET
	Attribute	Virtual landmarks
	Adjacent segments	{‘$l_{\mathrm{ET}}l_{s0}$’:{‘semantics’: ‘Go left’, ‘distance’: 9.12, ‘direction’: ‘West-South’}}
	Direction information	‘West’
	Semantic description	∅
Stairs s0	Id	s0
	Attribute	Stairs
	Adjacent segments	{‘$l_{s0}l_{s1}$’:{‘semantics’: ‘Climb the steps’, ‘distance’: 0.99, ‘direction’: ‘South’}}
	Direction information	‘South’
	Semantic description	∅
Stairs s1	Id	s1
	Attribute	Stairs
	Adjacent segments	{ ‘$l_{s1}l_{u0}$’:{‘semantics’: ∅, ‘distance’: 5.05, ‘direction’: ‘South’}}
	Direction information	‘South’
	Semantic description	∅
Turn u0	Id	u0
	Attribute	Turn
	Adjacent segments	{‘$l_{u0}l_{r8}$’:{‘semantics’: [‘Go straight’, ‘Turn right’], ‘distance’: 19.13, ‘direction’: ‘West-North’}}
	Direction information	‘South - West’
	Semantic description	‘Turn right’
Door r8	Id	r8
	Attribute	Door
	Adjacent segments	{‘$l_{r8}l_{E}$’:{‘semantics’: ∅, ‘distance’: 2.03, ‘direction’: ‘North}}
	Direction information	‘North’
	Semantic description	‘Go into the door’

**Table 6:** Complete semantics of turn u0.

Name	Expression
Id	u0
Attribute	Turn
Adjacent segments	{‘$l_{u0}l_{r1}$’: {‘semantics’: ‘Turn left (at 1st door)’, ‘distance’: 5.19, ‘direction’: ‘West-South’}, ‘$l_{u0}l_{r2}$’: {‘semantics’: ‘Turn right (at 1st door)’, ‘distance’: 5.21, ‘direction’: ‘West-North’}, ‘$l_{u0}l_{r3}$’: {‘semantics’: ‘Turn left (at 2nd door)’, ‘distance’: 6.87, ‘direction’: ‘West-South’}, ‘$l_{u0}l_{r0}$’: {‘semantics’: ‘Turn right (at 2nd door)’, ‘distance’: 7.19, ‘direction’: ‘West-North’}, ‘$l_{u0}l_{r4}$’: {‘semantics’: ‘Turn right (at 3rd door)’, ‘distance’: 12.13, ‘direction’: ‘West-North’}, ‘$l_{u0}l_{r5}$’: {‘semantics’: ‘Turn left (at 3rd door)’, ‘distance’: 12.27, ‘direction’: ‘West-South’}, ‘$l_{u0}l_{r7}$’: {‘semantics’: ‘Turn left (at 4th door)’, ‘distance’: 14.11, ‘direction’: ‘West-South’}, ‘$l_{u0}l_{r6}$’: {‘semantics’: ‘Turn right (at 4th door)’, ‘distance’: 15.96, ‘direction’: ‘West-North’}, ‘$l_{u0}l_{r9}$’: {‘semantics’: ‘Turn left (at 5th door)’, ‘distance’: 17.75, ‘direction’: ‘West-South’}, ‘$l_{u0}l_{r8}$’: {‘semantics’: ‘Turn right (at 5th door)’, ‘distance’: 19.13, ‘direction’: ‘West-North’}, ‘$l_{u0}l_{r10}$’: {‘semantics’: ‘Go straight’, ‘distance’: 20.28, ‘direction’: ‘West’}, ‘$l_{u0}l_{s1}$’: {‘semantics’: ‘Turn left’, ‘distance’: 5.09, ‘direction’: ‘North}, ‘$l_{u0}l_{s2}$’: {‘semantics’: ‘Turn right’, ‘distance’: 2.84, ‘direction’: ‘South’}}
Direction information	‘South-West’, ‘North-West’, ‘East-South’, ‘East-North’
Landmark semantic	‘Turn right’, ‘Turn left’

**Table 7:** Classification accuracy.

Classifier	Accuracy	Accuracy
	(Sliding Windows)	(Event-Defined Windows)
DT	98.62%	98.69%
SVM	96.55%	97.73%
KNN	98.83%	98.95%

**Table 8:** Confusion matrix.

Actual Class	Predicted Class				Accuracy (%)
	Standing	Walking	Going up (or down) Stairs	Opening a Door	
Standing	284	0	0	10	96.60%
Walking	0	1981	3	0	99.85%
Going up (or down) stairs	0	4	787	0	99.49%
Opening a door	3	0	0	56	94.92%

**Table 9:** Landmark matching errors.

Landmark	Total	Wrong Match	Error Rate
Doors	24	1	4.17%
Stairs	96	0	0
Turns	63	1	1.59%

**Table 10:** Comparison with other localization systems.

Name	Zee	UnLoc	The Proposed Approach
Requirement	Floorplan	A door location	Floorplan, landmarks
Sensors	Acc., Gyro., Mag., (Wi-Fi)	Acc., Gyro., Mag., (Wi-Fi)	Acc., Gyro., Mag., Baro.
User participation	None	Some	Some
Accuracy	1–2 m	1–2 m	<1 m
Expression	Trajectory	Trajectory	Trajectory, semantic description
Extensibility	Wi-Fi RSS distribution	Landmark distribution	Semantic landmark model

**Table 11:** Semantic matching results.

Trajectory	Trajectory Segment	Semantic	Time Complexity	Numbers ^1^
Trajectory information	Segment 1 (red points)	‘Turn right’ (‘East-South’)	O(N)	5
	Segment 2 (blue points)	‘Go straight’ (‘South’)	O(7)	2
	Segment 3 (purple points)	‘Turn right’ (‘South-West’)	O(3)	1

^1^ The number of trajectories after semantic matching.

## References

[1] Deng Z., Wang G., Hu Y., Cui Y. (2016). Carrying Position Independent User Heading Estimation for Indoor Pedestrian Navigation with Smartphones. Sensors.

[2] Gezici S., Tian Z., Giannakis G.B., Kobayashi H., Molisch A.F., Poor H.V., Sahinoglu Z. (2005). Localization via ultra-wideband radios: A look at positioning aspects for future sensor networks. IEEE Signal Proc. Mag..

[3] Zhu W., Cao J., Xu Y., Yang L., Kong J. (2014). Fault-tolerant RFID reader localization based on passive RFID tags. IEEE Trans. Parallel Distrib. Syst..

[4] Deng Z., Xu Y., Ma L. (2012). Indoor positioning via nonlinear discriminative feature extraction in wireless local area network. Comput. Commun..

[5] Li H., Chen X., Jing G., Wang Y., Cao Y., Li F., Zhang X., Xiao H. (2015). An Indoor Continuous Positioning Algorithm on the Move by Fusing Sensors and Wi-Fi on Smartphones. Sensors.

[6] Guinness R. (2015). Beyond Where to How: A Machine Learning Approach for Sensing Mobility Contexts Using Smartphone Sensors. Sensors.

[7] Incel O.D., Kose M., Ersoy C. (2013). A Review and Taxonomy of Activity Recognition on Mobile Phones. BioNanoScience.

[8] Shoaib M., Bosch S., Incel O., Scholten H., Havinga P. (2015). A Survey of Online Activity Recognition Using Mobile Phones. Sensors.

[9] Lara O.D., Labrador M.A. (2013). A Survey on Human Activity Recognition using Wearable Sensors. IEEE Commun. Surv. Tutor..

[10] Yang Z., Wu C., Zhou Z., Zhang X., Wang X., Liu Y. (2015). Mobility increases localizability: A survey on wireless indoor localization using inertial sensors. ACM Comput. Surv. (CSUR).

[11] Deng Z., Wang G., Hu Y., Wu D. (2015). Heading Estimation for Indoor Pedestrian Navigation Using a Smartphone in the Pocket. Sensors.

[12] Paucher R., Turk M. Location-based augmented reality on mobile phones. Proceedings of the 2010 IEEE Computer Society Conference on Computer Vision and Pattern Recognition Workshops (CVPRW).

[13] Kang W., Han Y. (2015). SmartPDR: Smartphone-Based Pedestrian Dead Reckoning for Indoor Localization. IEEE Sens. J..

[14] Alzantot M., Youssef M. UPTIME: Ubiquitous pedestrian tracking using mobile phones. Proceedings of the 2012 IEEE Wireless Communications and Networking Conference (WCNC).

[15] Chen Z., Zou H., Jiang H., Zhu Q., Soh Y., Xie L. (2015). Fusion of WiFi, Smartphone Sensors and Landmarks Using the Kalman Filter for Indoor Localization. Sensors.

[16] Evennou F., Marx F. (2006). Advanced integration of WiFi and inertial navigation systems for indoor mobile positioning. Eurasip J. Appl. Signal Process..

[17] Waqar W., Chen Y., Vardy A. Incorporating user motion information for indoor smartphone positioning in sparse Wi-Fi environments. Proceedings of the 17th ACM international conference on Modeling, analysis and simulation of wireless and mobile systems).

[18] Huang Q., Zhang Y., Ge Z., Lu C. Refining Wi-Fi Based Indoor Localization with Li-Fi Assisted Model Calibration in Smart Buildings. Proceedings of the International Conference on Computing in Civil and Building Engineering.

[19] Hernández N., Ocaña M., Alonso J.M., Kim E. (2017). Continuous Space Estimation: Increasing WiFi-Based Indoor Localization Resolution without Increasing the Site-Survey Effort. Sensors.

[20] Li F., Zhao C., Ding G., Gong J., Liu C., Zhao F. A reliable and accurate indoor localization method using phone inertial sensors. Proceedings of the 2012 ACM Conference on Ubiquitous Computing.

[21] Xiao Z., Wen H., Markham A., Trigoni N. Lightweight map matching for indoor localisation using conditional random fields. IPSN-14 Proceedings of the 13th International Symposium on Information Processing in Sensor Networks.

[22] Rai A., Chintalapudi K.K., Padmanabhan V.N., Sen R. Zee: Zero-effort crowdsourcing for indoor localization. Proceedings of the 18th Annual International Conference on Mobile Computing and Networking.

[23] Leppäkoski H., Collin J., Takala J. (2013). Pedestrian navigation based on inertial sensors, indoor map, and WLAN signals. J. Signal Process. Syst..

[24] Wang H., Lenz H., Szabo A., Bamberger J., Hanebeck U.D. WLAN-based pedestrian tracking using particle filters and low-cost MEMS sensors. Proceedings of the 4th Workshop on Positioning, Navigation and Communication (WPNC’07).

[25] Wang H., Sen S., Elgohary A., Farid M., Youssef M., Choudhury R.R. No need to war-drive: unsupervised indoor localization. Proceedings of the 10th International Conference on Mobile Systems, Applications, and Services.

[26] Constandache I., Choudhury R.R., Rhee I. Towards mobile phone localization without war-driving. Proceedings of the 29th Conference on Computer Communications.

[27] Anagnostopoulos C., Tsetsos V., Kikiras P. OntoNav: A semantic indoor navigation system. Proceedings of the 1st Workshop on Semantics in Mobile Environments (SME’05).

[28] Kolomvatsos K., Papataxiarhis V., Tsetsos V. (2009). Semantic Location Based Services for Smart Spaces.

[29] Tsetsos V., Anagnostopoulos C., Kikiras P., Hadjiefthymiades S. (2006). Semantically enriched navigation for indoor environments. Int. J. Web Grid Serv..

[30] Park J., Teller S. (2014). Motion Compatibility for Indoor Localization.

[31] Harle R. (2013). A Survey of Indoor Inertial Positioning Systems for Pedestrians. IEEE Commun. Surv. Tutor..

[32] Sun Z., Mao X., Tian W., Zhang X. (2008). Activity classification and dead reckoning for pedestrian navigation with wearable sensors. Meas. Sci. Technol..

[33] Kappi J., Syrjarinne J., Saarinen J. MEMS-IMU based pedestrian navigator for handheld devices. Proceedings of the 14th International Technical Meeting of the Satellite Division of the Institute of Navigation (ION GPS 2001).

[34] Kang W., Nam S., Han Y., Lee S. Improved heading estimation for smartphone-based indoor positioning systems. Proceedings of the 2012 IEEE 23rd International Symposium on Personal Indoor and Mobile Radio Communications (PIMRC).

[35] Gusenbauer D., Isert C., Krösche J. Self-contained indoor positioning on off-the-shelf mobile devices. Proceedings of the 2010 International Conference on Indoor Positioning and Indoor Navigation (IPIN).

[36] Preece S.J., Goulermas J.Y., Kenney L.P., Howard D., Meijer K., Crompton R. (2009). Activity identification using body-mounted sensors—A review of classification techniques. Physiol. Meas..

[37] Banos O., Galvez J., Damas M., Pomares H., Rojas I. (2014). Window Size Impact in Human Activity Recognition. Sensors.

[38] Wu W., Dasgupta S., Ramirez E.E., Peterson C., Norman G.J. (2012). Classification accuracies of physical activities using smartphone motion sensors. J. Med. Internet Res..

[39] Shoaib M., Bosch S., Incel O., Scholten H., Havinga P. (2016). Complex Human Activity Recognition Using Smartphone and Wrist-Worn Motion Sensors. Sensors.

[40] Damaševičius R., Vasiljevas M., Šalkevičius J., Woźniak M. (2016). Human Activity Recognition in AAL Environments Using Random Projections. Comput. Math. Methods Med..

